# Trace Sample Proteome Quantification by Data-Dependent Acquisition without Dynamic Exclusion

**DOI:** 10.1021/acs.analchem.3c03357

**Published:** 2023-11-30

**Authors:** Ci Wu, Jiao Lei, Fei Meng, Xingyao Wang, Cassandra J. Wong, Jiaxi Peng, Ge Lin, Anne-Claude Gingras, Junfeng Ma, Shen Zhang

**Affiliations:** † Department of Oncology, Lombardi Comprehensive Cancer Center, Georgetown University Medical Center, Georgetown University, Washington D.C. 20007, United States; ‡ Clinical Research Center for Reproduction and Genetics in Hunan Province, Reproductive and Genetic Hospital of CITIC-XIANGYA, Changsha, Hunan 410000, China; § National & Local Joint Engineering Laboratory of Animal Peptide Drug Development, College of Life Sciences, Hunan Normal University, Changsha, Hunan 410081, China; ∥ Lunenfeld-Tanenbaum Research Institute, Toronto, Ontario M5G 1X5, Canada; ⊥ Department of Chemistry, University of Toronto, Toronto, Ontario M5S 3G9, Canada; ▲ Department of Molecular Genetics, University of Toronto, Toronto, Ontario M5G 1X8, Canada

## Abstract

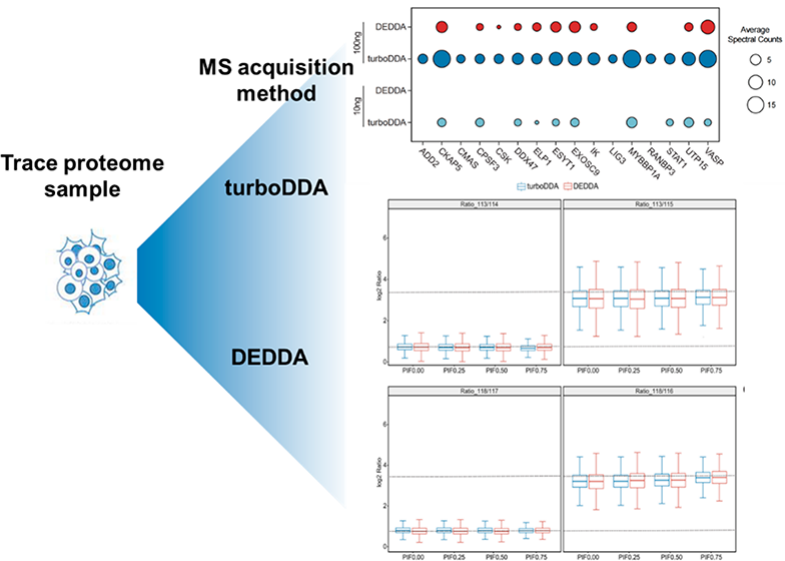

Despite continuous technological improvements in sample preparation, mass-spectrometry-based proteomics for trace samples faces the challenges of sensitivity, quantification accuracy, and reproducibility. Herein, we explored the applicability of turboDDA (a method that uses data-dependent acquisition without dynamic exclusion) for quantitative proteomics of trace samples. After systematic optimization of acquisition parameters, we compared the performance of turboDDA with that of data-dependent acquisition with dynamic exclusion (DEDDA). By benchmarking the analysis of trace unlabeled human cell digests, turboDDA showed substantially better sensitivity in comparison with DEDDA, whether for unfractionated or high pH fractionated samples. Furthermore, through designing an iTRAQ-labeled three-proteome model (i.e., tryptic digest of protein lysates from yeast, human, and *E. coli*) to document the interference effect, we evaluated the quantification interference, accuracy, reproducibility of iTRAQ labeled trace samples, and the impact of PIF (precursor intensity fraction) cutoff for different approaches (turboDDA and DEDDA). The results showed that improved quantification accuracy and reproducibility could be achieved by turboDDA, while a more stringent PIF cutoff resulted in more accurate quantification but less peptide identification for both approaches. Finally, the turboDDA strategy was applied to the differential analysis of limited amounts of human lung cancer cell samples, showing great promise in trace proteomics sample analysis.

## Introduction

Precision proteome quantification provides insights into protein composition and function within biological systems, driving clinical research and molecular medicine.^1^ Given the limited availability of many important biological samples, the development of a trace sample proteomic analysis method is crucial.^2^ Great efforts have been made in recent years to reduce sample requirements for proteomic analyses,^3−9^ while accurate and reproducible quantification of trace samples remains a challenge in many scenarios. Several integrated approaches have been developed recently, enabling sensitive and robust proteomic quantification of low-input samples, including label-free quantification and stable isotope label-based quantification.^10−12^ Chemical tagging with isobaric tags (e.g., iTRAQ or TMT labeling) is widely used across multiple application fields for its sample multiplexing capability that permits increases in sample input and throughput.^13−15^ The accuracy of quantitative data obtained by means of this approach can be adversely affected by interference from coeluted and cofragmented ions for the target precursors.^16^

Mass spectrometry (MS) detects proteomics samples most often by using data-dependent acquisition (DDA) with dynamic exclusion (DE) (DEDDA), which excludes reselection of a peptide within a given time window.^17^ Although dynamic exclusion renders more identifications and a higher possibility to find low-abundant proteins, stochastic and irreproducible precursor ion selection often limits the accurate quantification analysis of trace samples, especially for MS/MS-based quantification. This is because a peptide is usually triggered for fragmentation only once due to dynamic exclusion, and the fragment ions used for quantification only reflect the peptide abundances at that given time point.^18^

By turning off the “dynamic exclusion” function, when the scan speed is fast enough to ensure adequate PSM as for one peptide, the DDA mode has the potential of overcoming the problems of stochastic and irreproducible precursor ion selection and under-sampling, thereby increasing the chances of successful identification of low-abundance peptides.^17^ Previously, we developed a data-dependent acquisition without dynamic exclusion method (which we termed turboDDA) for bulk proteomics quantification, resulting in improved accuracy and reproducibility.^19−21^ Given the critical challenges of trace proteomics, we explored the feasibility and applicability of turboDDA. In this study, we systematically optimized the acquisition parameters of turboDDA specifically for minute amounts of samples. We then evaluated quantification interference, accuracy, reproducibility, and the impact of PIF (precursor intensity fraction) function on different approaches (turboDDA and DEDDA). Finally, this turboDDA strategy was successfully applied to trace proteomics quantification of human lung cancer cells.

## Experimental Section

### Sample Preparation of iTRAQ-Labeled Three-Proteome Mixture

We used 8 plex iTRAQ reagents to build a three-proteome peptide mixture sample from the digests of human K562 cells, yeast, and *E. coli* (Figure S1). In comparison to model samples that was used to study the interference effect reported previously,^22,23^ our approach allows direct observation of interference from human and *E. coli* peptide ions from channels 119 and 121. The detailed labeling ratios of each channel for each sample are shown in the Supporting Information.

### Liquid Chromatography and Tandem Mass Spectrometry

Sample of human digests and iTRAQ-labeled three-proteome mixtures were analyzed by TripleTOF 6600 instrument (AB SCIEX). Data were acquired with DEDDA and turboDDA mode. As a comparison, a Thermo Fisher Orbitrap Eclipse Tribrid mass spectrometer (MS) was also used for data acquisition. Details for method optimization and application parameters are described in the Supporting Information.

### Data Analysis

Mass spectrometry data were stored, searched, and analyzed using the ProHits laboratory information management system platform,^24,25^ Proteome Discoverer, MaxQuant, and ProteinPilot, with details shown in the Supporting Information.

## Results and Discussion

**Figure 1:**
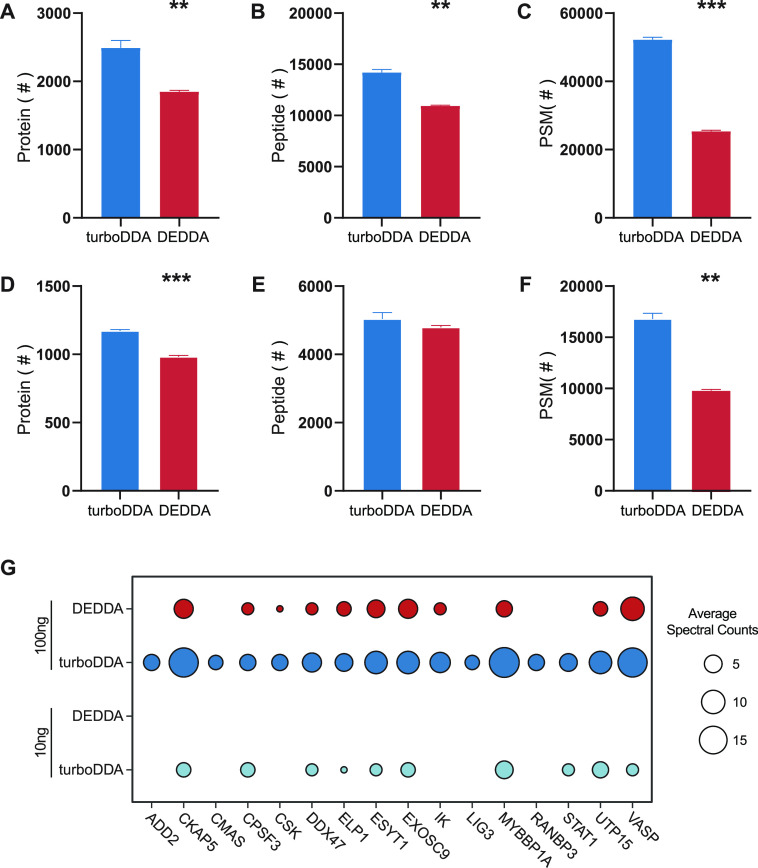
Identification results of trace amounts of sample by turboDDA and DEDDA. The number of proteins, peptides, and PSMs identified were obtained by turboDDA and DEDDA methods from 100 ng (A, B, and C) and 10 ng (D, E, and F) of human K562 cell digests (*n* = 3, average). *p < 0.05, **p < 0.01, and ***p < 0.001, by two-tailed unpaired Student’s *t*-test. (G) Spectral counts of a subset of proteins identified only by turboDDA in 10 ng of the digest or 100 ng of the digest.

To achieve the best performance, we systematically optimized the acquisition parameters for turboDDA and DEDDA for the analysis of trace samples (Table S1). In brief, we evaluated parameters for dynamic exclusion, top MS/MS candidate number, precursor intensity threshold, and MS/MS accumulation time. Then the performance of the optimized turboDDA and that of the optimized DEDDA method was compared from four aspects (i.e., protein number, peptide number, PSMs, and MS/MS number). As can be seen from Figure 1, turboDDA shows great priority in the identification of minute amounts of samples. For 100 ng of sample, turboDDA identified more proteins (2492 vs 1833, *p*-value = 0.007), peptides (14,192 vs 10,996, *p*-value = 0.002), and PSMs (52,034 vs 25,199, *p*-value = 0.00004) than the DEDDA method. Even with a decreased sample amount of 10 ng, turboDDA yielded more proteins (1171 vs 973, *p*-value = 0.00002), peptides (5037 vs 4782, *p*-value = 0.149), and PSMs (16,791 vs 9682, *p*-value = 0.001). Overlap of the identification results on protein and peptide levels for turboDDA and DEDDA suggest that turboDDA covered most identifications from DEDDA (Figure S2). In addition, because turboDDA acquired the top 100 MS/MS candidate ions while DEDDA only acquired the top 10 MS/MS candidate ions in parameter settings, turboDDA could acquire much more MS/MS spectra than DEDDA (Figure S3 and Table S2). To investigate the characteristics of the peptides and proteins identified by turboDDA and DEDDA, the grand average of hydropathy (GRAVY) value, isoelectric point (pI), and molecular weight (MW) distribution of both methods, and the abundance distribution for the commonly and uniquely identified proteins by both methods were shown in Figure S4. No obvious bias could be found from these results for proteins and peptides identified by turboDDA. Moreover, by referencing to a large scale proteomics study of K562 cells,^26^ many middle- to low-abundant proteins in the data set were only identified by the turboDDA method (Figure 1G). Representative mass spectra of three peptides from low-abundance proteins (Figure S5) further show an overall improved quality of MS/MS spectra acquired by the turboDDA method. For complex sample analysis, fractionation was usually necessary to obtain deeper identification. Herein, the performance of turboDDA for K562 cell digests with high pH fractionation was also evaluated in comparison with that of DEDDA. As shown in Table S3, for 250 ng cell digests, the number of proteins (5459 vs 4609), peptides (43,742 vs 32,687), and PSMs (350,222 vs 148,347) identified by turboDDA were all higher than the DEDDA acquisition method. When only 25 ng of sample was used for fractionation, turboDDA still provided better identification performance than DEDDA for proteins (3794 vs 2792), peptides (15,780 vs 13,165), and PSMs (95,831 vs 48,364). All of these results show that turboDDA is superior in protein and peptide identification. Since the conventional DEDDA method only collects a single scan for many peptides (which decreases identification chances from sampling nearer the apex), this may lead to inadequate identification of low abundant proteins, especially in trace samples. In comparison, turboDDA allows for multiple scans for most peptide sequences, increasing the chances of scanning at the peak apex of the peptide and thus the successful identification. Besides the TripleTOF instrument, we tested turboDDA and DEDDA with low input amounts (10 ng and 1 ng K562 digests) on an Orbitrap Eclipse Tribrid mass spectrometer (Figure S6). Very encouragingly, turboDDA also yielded better identification and improved quantification reproducibility than DEDDA on this relatively slower-scanning instrument.

**Figure 2:**
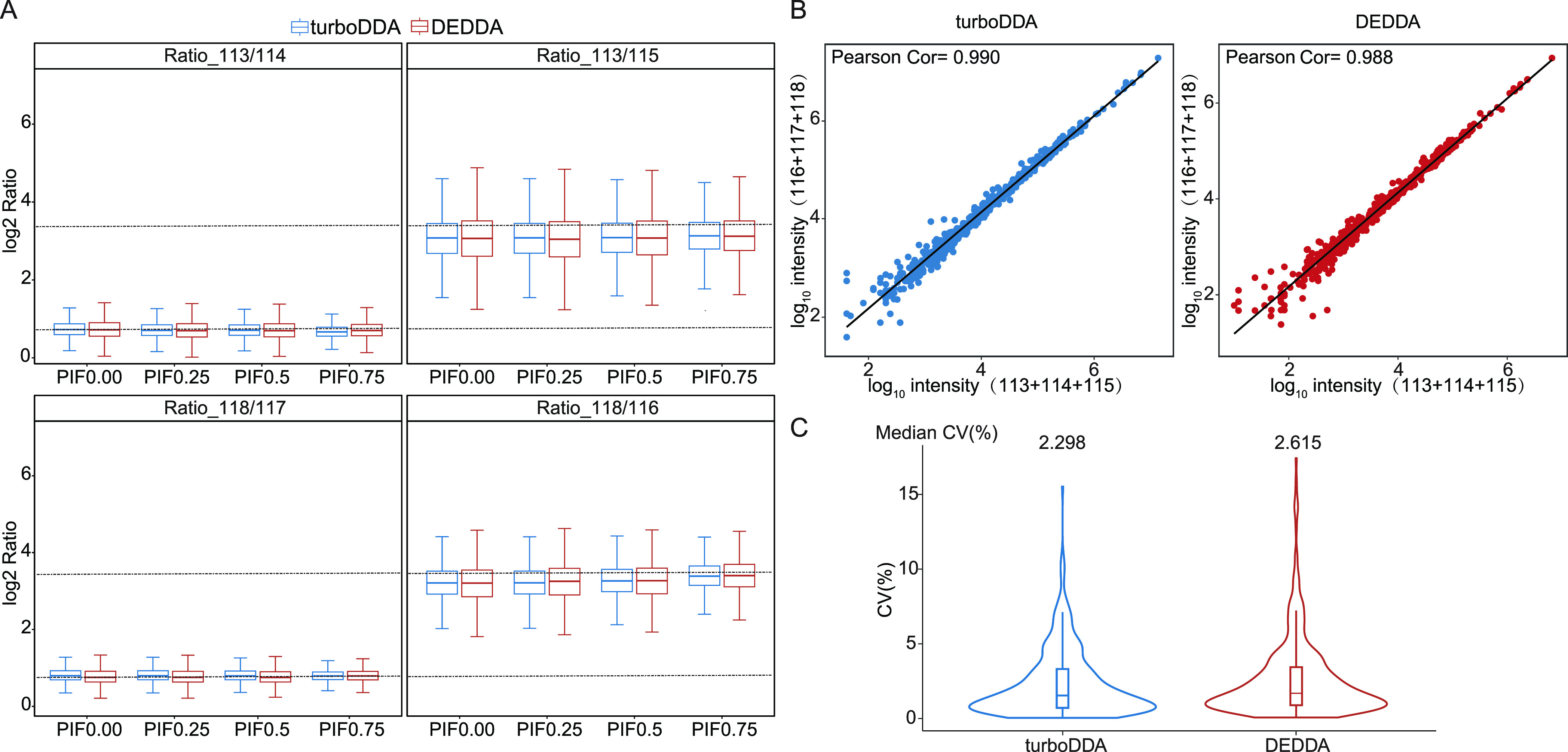
Quantification performances of turboDDA and DEDDA. (A) Comparison on the quantitative accuracy of the turboDDA and DEDDA for the analysis of iTRAQ-labeled three-proteome mixture. (B) The correlations between the iTRAQ intensity of channels with and without interference for turboDDA and DEDDA. (C) The coefficients of variation (CV) distribution of iTRAQ intensities from three injections for turboDDA and DEDDA.

The influence of the acquisition mode of turboDDA and DEDDA on quantification was evaluated by using the iTRAQ-labeled three-proteome mixture (Figure S1). We first compared the quantitative accuracy of turboDDA and DEDDA (Figure 2A). When no Precursor Intensity Fraction (PIF) cutoff was applied, the median reporter ion ratios of turboDDA were 1.69 and 8.43 for 118/117 and 118/116, with SD values of 0.48 and 0.65, respectively, while the corresponding ratios with the DEDDA method were 1.66 and 8.39, with SD values of 0.55 and 0.73, respectively. For channels with *E. coli* and human cell digests as interference (channels 113, 114 and 115), the ratios of turboDDA turned into 1.66 (113/114) and 7.99 (113/115), with SD values of 0.49 and 0.77, and the corresponding ratios of DEDDA were 1.65 and 7.92, with SD values of 0.57 and 0.84, respectively. These results show that turboDDA yield better quantification accuracy than DEDDA no matter with or without interference, and more serious ratio compression could be observed with interference compared to that without interference.

Precursor Intensity Fraction (PIF) filter is a strategy used in MaxQuant to improve the accuracy of multiplex quantification.^27^ By setting a PIF threshold, MS/MS spectra whose precursor with a specified amount of interference in the isolation window can be filtered out to improve the accuracy of multiplex quantification. PIF filtering is commonly used in data-dependent acquisition (DDA) methods to reduce chimera spectra from the precursor level. Here, we sought to investigate the impact of PIF filtering on spectral purity and iTRAQ quantification accuracy of turboDDA and DEDDA. Theoretically, many more acquisition points could be acquired with turboDDA, which is prone to increase the cofragmentation probability and generate more chimera spectra. However, from the comparison of PIF value distribution between turboDDA and DEDDA (Figure S7), we found the median PIF value of turboDDA (0.73) is even higher than that of DEDDA (0.71), indicating more acquisition points in turboDDA did not bring more cofragmentation in general. Other than PIF, the three proteome models allow us to evaluate the spectral purity in the MS/MS level by interference-free index (IFI), which is calculated from the ratio of the reporter ions intensity of the contaminating channels (119 and 121) to all eight channels (Figure S8A). For quantified yeast peptides, an IFI value close to 1 would indicate that the ion intensity is mostly derived from yeast peptides with minimal contamination from human and *E. coli* peptides. We defined the yeast peptides with an IFI greater than 0.95 as interference-free identifications: these represented 42.27% (2282/5399) in turboDDA versus 39.44% (2102/5330) in DEDDA when no PIF filtering was applied. With the PIF threshold increased, the median IFI value increased from 0.941 (PIF = 0.00) to 0.956 (PIF = 0.75) for turboDDA and from 0.937 (PIF = 0.00) to 0.956 (PIF = 0.75) for DEDDA (Figure S8B), and the corresponding quantification accuracy was also improved (Figure 2A), as demonstrated by the SD value decreasing from 0.49 to 0.43 for the 113/114 ratio in turboDDA results. On one hand, these results indicated again that the turboDDA method would not only cause more cofragmentation but also slightly improved spectral purity compared with the DEDDA method. On the other hand, PIF filtering could indeed increase spectral purity (Figure S8B) and quantitative accuracy (Figure 2A) for multiplex labeled samples. However, although a more stringent PIF cutoff benefits quantification, the yeast peptide identification number was significantly reduced (Figure S8C), especially when the PIF cutoff increased from 0.50 to 0.75. Besides, compared to DEDDA, no matter which PIF filtering threshold was used, turboDDA identified more peptides. The advantage of turboDDA in identification compared with DEDDA for this iTRAQ-labeled three-proteome model is not so obvious as in the identification of 10 ng and 100 ng unlabeled K562 digests, probably because the target sample (yeast) has less complexity (consistent with our previous work^20,21^). Considering that more acquisition points in turboDDA might cause more serious stochasticity in acquisition compared with DEDDA, we evaluated the inconsistency between the intensity of channels with and without interference by calculating the Pearson coefficient between the intensity of clean channels (116–118) and the intensity of channels with interference (113–115). As shown in Figure 2B, the Pearson correlation coefficient is 0.990 for turboDDA, which is comparative with that of DEDDA (0.988). Furthermore, the reproducibility of the quantification was evaluated by the coefficients of variation (CV) among the intensities of quantified peptides from different injections (Figure 2C). The median CV of turoDDA is lower than that of DEDDA (2.298 vs 2.615), demonstrating better reproducibility.

The study of lung cancer cells is of significant importance in understanding the biology, diagnosis, and treatment of lung cancer. It helps in identifying the genetic and molecular changes that occur in cancer cells, which can lead to the development of new targeted therapies. Although A549 and Calu-6 are both metastatic lung cancer cells, their metastatic ability is different; the metastatic ability of A549 cells is relatively weak, mainly manifested as weak migration ability and low invasion ability. While the metastatic ability of Calu6 cells is relatively strong, it manifested as highly migratory and invasive ability.^28,29^ Further in-depth research of these two kinds of lung cancer cells will help in understanding the metastasis of lung cancer and the development of potentially new therapies.

**Figure 3:**
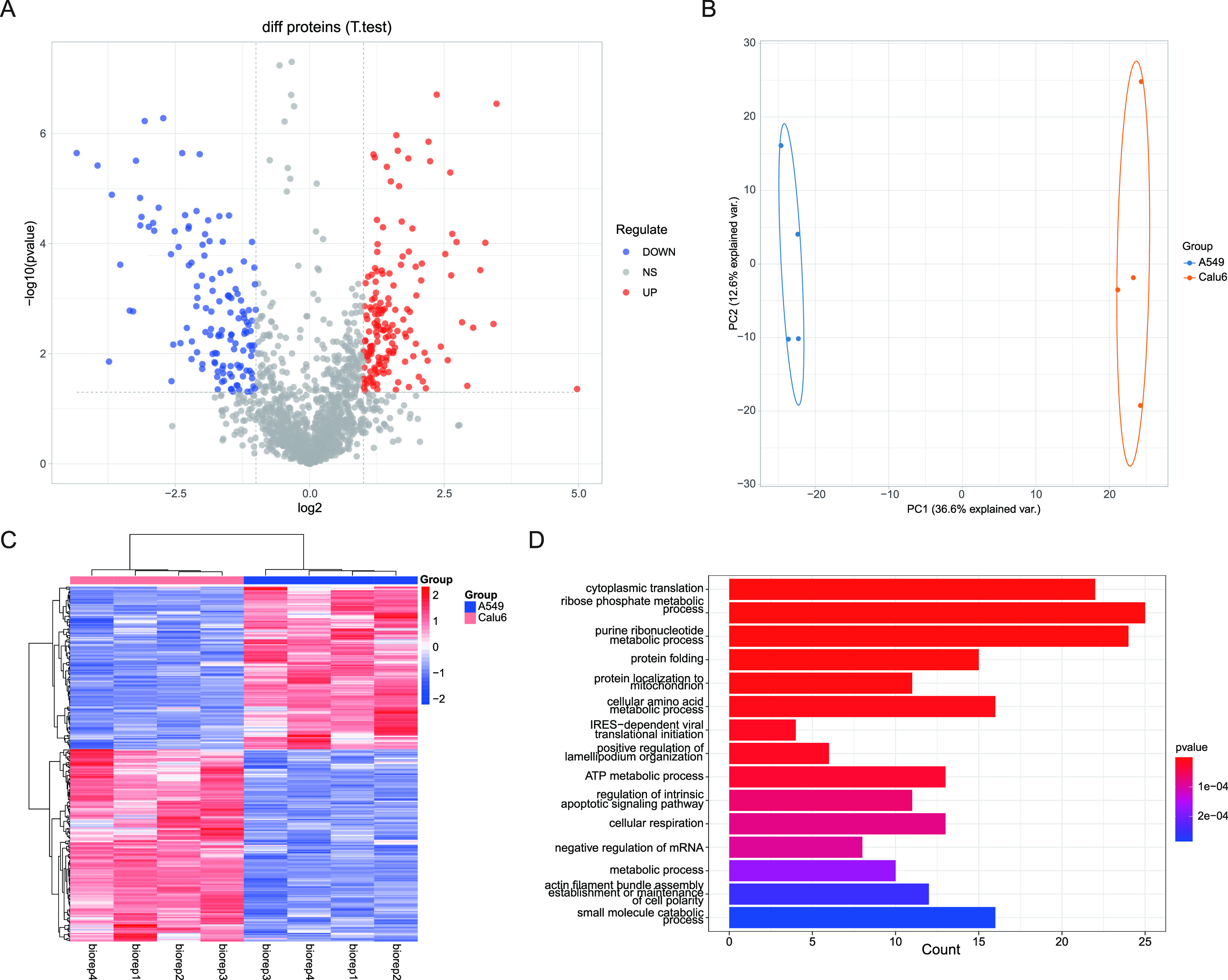
Differential analysis of human lung cancer H/L cell line A549 and Calu6. (A) Volcano plot of the global quantification of proteins in A549 and Calu6 cell lines. (B) Principle component analysis of the proteins in A549 and Calu6 cell group. (C) Heatmap of the proteins differentially expressed between A549 and Calu6. (D) Gene ontology analysis of the biological processes of differential expression proteins.

Herein, a total of 1688 proteins were quantified from lung cancer cells of A549 and calu6, including 290 differentially expressed proteins in these two groups (Figure 3A). Among them, 157 were upregulated in the low metastatic A549 cell line, and 133 were upregulated in the highly metastatic Calu6 cell line. These differential proteins are mostly related to the occurrence and metastasis of tumors. Cystatin-C encoded by the CST3 gene is one of the most differentially expressed proteins in A549 cell. Studies have shown that CST3 was a potential causal gene for airflow obstruction in the lung.^30^ OCIAD2 (ovarian carcinoma immunoreactive antigen domain 2), which was a biomarker of early lung adenocarcinoma, was also identified upregulated in Calu6 cell.^31^ Therefore, further research of these differential proteins may be beneficial for the discovery of potential biomarkers and therapeutic targets for lung cancer. Furthermore, principal component analysis (PCA) was performed for the data sets (Figure 3B). The first component (PC1) clearly differentiates the A549 group from Calu-6. There are some variations on the second component (PC2), which may be caused by the perturbation due to the reproducibility of the sample preparation. The distinct proteome profiles indicate that they may serve as potential markers for lung cancer. Unsupervised clustering of the quantified proteins also resulted in the distinction of lowly migratory tumor cell A549 from highly invasive cell Calu-6 (Figure 3C). As shown in Figure 3D, genome ontology (GO) analysis showed that many significant regulated proteins have close relationships with the biological processes of cytoplasmic translation, ribose phosphate metabolism, purine ribonucleotide metabolism, and protein folding. Molecular functional analysis shows that those proteins are prominently enriched in nucleotide biosynthetic and metabolic processes, with a high portion of binding proteins including cadherin binding, GTP binding, and unfold protein binding (Figure S9A). In addition, the protein interaction network indicates that unregulated proteins are highly clustered in focal adhesion, pigment granule, primary lysosome, and cytosolic ribosome (Figure S9B).

## Conclusion

Here, we report the development and application of an efficient turboDDA method for the proteomics analysis of trace samples. It shows substantially increased sensitivity than the regular DEDDA method for sample amounts as low as 1 ng. Besides on fast-scanning TOF instruments, turboDDA also yielded higher identifications than DEDDA on slow-scanning Orbitrap instruments for trace amounts of samples. Improved quantification accuracy and reproducibility of the turboDDA approach were also obtained on the iTRAQ labeling quantification in comparison with that of the DEDDA method. Furthermore, this turboDDA strategy was successfully applied to proteomics quantification of a trace amount of human lung cancer cells. We believe that isobaric-labeling-based quantitation combined with turboDDA has great potential for the analysis of clinical samples, for which high throughput, high accuracy, and high sensitivity are essential. Of note, as turboDDA is fully compatible with other advanced techniques such as ion mobility and MS3 fragmentation, we propose that combining turboDDA with these techniques may result in further improved accuracy in proteomic quantification. Moreover, with the rapid development of data independent acquisition (DIA) methods and their successful applications in trace/single cell proteome analysis, the comparison between turboDDA and different DIA methods should be of interest to explore.

## Data Availability

All data from the unlabeled K562 digest and iTRAQ-labeled samples were deposited in the ProteomeXchange Consortium (https://proteomecentral.proteomexchange.org) via the PRIDE partner repository with identifier PXD042252, PXD042385, and PXD042385, respectively.

## Supporting Information Available

Experimental section of chemicals and materials, sample preparation, liquid chromatography and mass spectrometry, and data analysis; additional figures and tables (PDF)
