# Supplementary material for: Trace Sample Proteome Quantification by Data-Dependent Acquisition without Dynamic Exclusion
Source: Anal Chem. 2023 Nov 30;95(49):17981–7. doi: 10.1021/acs.analchem.3c03357 (PMC10719888; doi:10.1021/acs.analchem.3c03357)

*Supporting Information*

# Trace Sample Proteome Quantification by Data-Dependent Acquisition without Dynamic Exclusion

Ci Wu[1,#], Jiao Lei[2,#], Fei Meng[2], Xingyao Wang[3], Cassandra J. Wong[4], Jiaxi Peng[5], Ge Lin[2], Anne-Claude Gingras[4,6], Junfeng Ma[1]*, Shen Zhang[2]*

[1]Department of Oncology, Lombardi Comprehensive Cancer Center, Georgetown University Medical Center, Georgetown University, Washington D.C. 20007, United States

[2]Clinical Research Center for Reproduction and Genetics in Hunan Province, Reproductive and Genetic Hospital of CITIC-XIANGYA, Changsha, Hunan 410000, China

[3]National & Local Joint Engineering Laboratory of Animal Peptide Drug Development, College of Life Sciences, Hunan Normal University, Changsha, Hunan 410081, China

[4]Lunenfeld-Tanenbaum Research Institute, Toronto, Ontario M5G 1X5, Canada

[5]Department of Chemistry, University of Toronto, Toronto, Ontario M5S 3G9, Canada

[6]Department of Molecular Genetics, University of Toronto, Toronto, Ontario M5G 1X8, Canada

[#]These authors contributed equally to this work

*Contact details for the corresponding authors:

Shen Zhang
E-mail: szhang231@126.com
Address: 567 Tongzipo West Road, Clinical Research Center for Reproduction and Genetics in Hunan Province, Reproductive and Genetic Hospital of CITIC-XIANGYA, Changsha, Hunan 410000, China

Junfeng Ma
Email: Junfeng.Ma@georgetown.edu
Address: Lombardi Comprehensive Cancer Center, Georgetown University Medical Center, 3900 Reservior Road NW, Washington DC, USA 20007

# Table of Content


| Description | Page No. |
|---|---|
| **Experimental section** | S-3 |
| **Table S1.** Optimization of acquisition parameters for the turboDDA and DEDDA methods | S-9 |
| **Table S2.** Total MS/MS spectra for turboDDA and DEDDA | S-10 |
| **Table S3.** Identified proteins number from 250 ng and 25 ng K562 cell digests by turboDDA and DEDDA methods | S-11 |
| **Figure S1.** Three-proteome model to simulate complex samples | S-12 |
| **Figure S2.** Venn diagrams on protein and peptide level for turboDDA and DEDDA | S-13 |
| **Figure S3.** Acquired MS/MS spectra and identified MS/MS spectra for DEDDA and turboDDA along retention time | S-14 |
| **Figure S4.** Characteristics of the peptides identified by DEDDA and turboDDA and the abundance distribution for the commonly identified and unique identified proteins by turboDDA and DEDDA | S-15 |
| **Figure S5.** Typical spectra of peptides acquired by turboDDA and DEDDA | S-16 |
| **Figure S6.** Trace amount of sample analysis by turboDDA and DEDDA on Thermo Orbitrap Eclipse instrument | S-17 |
| **Figure S7.** PIF value distribution of iTRAQ labeled three proteome mixtures for turboDDA and DEDDA | S-18 |
| **Figure S8.** Impact of PIF on interference free index (IFI) distribution and quantified peptide number for turboDDA and DEDDA | S-19 |
| **Figure S9.** GO analysis of the molecular functions and the interaction network of differential proteins from lung cancer cell lines | S-20 |

## Experimental Section

### Chemicals and Reagents

DTT (1,4-dithiothreitol) was purchased from Sigma-Aldrich (St. Louis, MO). Triethylammonium bicarbonate (TEAB stock) buffer (1 M, pH 8.4~8.6) was ordered from Fluka. Iodoacetamide (IAA) was ordered from VWR. Formic acid (FA, LC/MS grade) and acetonitrile (ACN, LC/MS grade) were purchased from Fisher Scientific (Waltham, MA). Nano UPLC mobile phase A: 0.1% formic acid in water (LC/MS grade) and mobile phase B: 100% ACN 0.1% formic acid (LC/MS grade) were ordered from Honeywell. S-Trap micro columns were purchased from Protifi (Huntington, NY, USA). Trypsin (MS grade) were purchased from Promega (Madison, WI, USA).

Digests of Human K562 cells (Cat#V6951) and yeast (Cat#V7461) were purchased from Promega (Madison, WI, USA). *E. coli* (Cat#186003196) digest was purchased from Waters. 8 plex iTRAQ reagents (Cat#4390812) were purchased from Sciex. The spin columns (Cat#89852) were purchased from Thermo Scientific.

### Cell Culture

The A549 and Calu-6 cells were grown in Dulbecco's modified Eagle's medium (DMEM) supplemented with 10% fetal bovine serum (FBS) and 1% penicillin/streptomycin. Cells were maintained in a 37 °C incubator with 5% $CO_2$. After treatment with 0.25% (w/v) trypsin/EDTA treatment, cells were washed once with 10 mL cold PBS. The cell suspension was centrifuged at 1200 rpm for 2 min at 4 °C, with the cell pellet kept at -80 °C before analysis.

### Protein extraction and digestion

Cell pellets were suspended in 50 µL cell lysis buffer (5% SDS, 1x protease inhibitor cocktail, 50 mM TEAB) by pipetting up and down. The cell suspension was then sonicated with a probe-tip sonicator for 2 pulses (10 sec on and 20 sec off for each pulse) on ice. The cell lysates were centrifuged at 13000 g for 15 min at 4 °C, with the supernatant transferred into a new 1.5 mL tube. Extracted proteins were processed with the suspension trapping (S-Trap) method as reported before.[2] Briefly, proteins were first reduced in 20 mM DTT by heating at 95 °C for 10 min. After cooling down, iodoacetamide was added to a final concentration of 40 mM for alkylation in darkness at room temperature for 30 min. The cell lysate solution was acidified by aqueous phosphoric acid (a final concentration of ~1.2% phosphoric acid) and diluted by six volumes of

the S-Trap buffer (90% aqueous methanol in 100 mM TEAB, pH 7.1). The acidified mixture was transferred onto a midi S-Trap column followed by centrifugation at 2000 g for 1 min. After washing with the S-Trap buffer three times, proteins on the column were digested with trypsin (an enzyme to substrate ratio of 1:50, w/w) at 37 °C overnight. The resulting peptides were eluted by adding 50 µL of 0.2% formic acid and 50% acetonitrile containing 0.2% formic acid subsequently. The elutes were combined and dried down with SpeedVac.

For proteome quantification, four biological repeats were processed for each cell line samples (A549 and Calu-6), and the peptides were labeled with iTRAQ 8-plex reagents as follows: four repeats of A549 digests were labeled with iTRAQ reagents 113, 114, 115, 116, and Calu-6 digests were labeled with 117, 118, 119 and 121, respectively. After labeling, all the eight digests were combined and dried with a lyophilizer. The mixture sample was fractioned with the high pH reversed-phase fractionation kit from Thermo Fisher Scientific (Cat# 84868) according to the manufacturer's instructions.

**Sample preparation of iTRAQ labeled three proteome mixture**

In brief, as shown in Figure S1, we labeled yeast peptides with all six reagents and mixed the differentially labeled peptides so that the ratio of reporter ions at channels from 113 to 115 was 10:6:1, and the ratio of reporter ions at channels from 116 to 118 was 1:6:10. We then labeled equal amounts of human peptides with iTRAQ reagents that generate reporter ions at m/z of 113, 114, 115 and 119 and labeled equal amounts of *E. coli* peptides with iTRAQ reagents that generate reporter ions at m/z of 113, 114, 115 and 121, respectively. After labeling, all aliquots were combined and the mixture was desalted using spin columns. We used this sample to measure the interference effect. Without interference, we expected ratios of channels 113 to 114, 114 to 115 and 113 to 115 to be equal to the ratios of channels 118 to 117, 117 to 116 and 118 to 116, respectively, for each yeast peptide ion selected for $MS^2$ analysis. Interference from human or/and *E. coli* peptide ions on yeast ions in channels 113, 114 and 115 was responsible for a leveling out of yeast reporter ion intensities so that ratios measured for channels 113, 114 and 115 were less than those measured for channels 116, 117 and 118. Furthermore, spiking human and *E. coli* digests into channels 119 and 121 makes these two channels can reflect the interference from human and *E. coli*.

**Liquid Chromatography and Mass Spectrometry**

The LC gradient for analysis of K562 cell digests started at 2% acetonitrile with 0.1% formic acid and increased to 35% acetonitrile over 90 min, followed by a 15 min wash at 80% acetonitrile and a 15 min equilibration at 2% acetonitrile, for a total of 120 min. Each sample was analyzed on a TripleTOF 6600 in DDA mode with and without DE. DDA with DE (DEDDA) consisted of one 250 ms MS1 TOF survey scan from 400–1250 Da followed by 10 × 100 ms $MS^2$ candidate ion scans from 100–1800 Da in high sensitivity mode. Only ions with a charge of 2+ to 5+ that exceeded a threshold of 300 cps were selected for $MS^2$, and former precursors were excluded for 7 s after one occurrence. For DDA mode without DE (turboDDA), precursors that exceeded a threshold of 100 cps were selected for MS2 and 100 × 30 ms MS2 candidate ion scans from 100–1800 Da were acquired in high sensitivity mode, using the same acquisition parameters otherwise. For both acquisition method, adjusted CE was used when analyzing iTRAQ labeled three proteome mixture. The turboDDA method was optimized by testing acquisition parameters, including the use of low resolution Q1 or unit resolution Q1; the precursor intensity threshold (100 cps or 300 cps); and the MS/MS accumulation time (30ms or 45ms); the same cycle time (3.25s) but different number of peptide ions selected for MS/MS fragmentation (top 50 or top 100) and different MS/MS accumulation time (60 ms or 30 ms). The DEDDA method was also optimized by testing several acquisition parameters, including the use of different dynamic exclusion time (7s or 20s); precursor exclusion after 1 time triggered or after 3 times triggered; different number of peptide ions selected for MS/MS fragmentation (top 10 or top 100) and different MS/MS accumulation time (100 ms or 30 ms).

For testing turboDDA and DEDDA methods on thermo instruments, a Thermo Fisher Orbitrap Eclipse Tribrid mass spectrometer (MS) equipped with a FAIMS Pro Interface was used to conduct experiments. K562 digests with different amounts (1ng or 10ng) were directly loaded to a 2 cm PEPMAP trap column on a Vanquish Neo UHPLC system (ThermoFisher Scientific). Besides, separation was performed on a 25 cm PepMap analytical column (ThermoFisher Scientific) with 3% to 38% buffer B (80% ACN, 0.1% formic acid) for 102 min and kept at 100% buffer B for 10min. FAIMS switched between CVs of −35 V, -45V and −65 V. MS1 spectra were obtained in the Orbitrap (resolution: 60k; AGQ target: standard; MaxIT: Auto; RF lens: 50%; mass range: 350 to 1,500). For DEDDA method, dynamic exclusion was used for 20 s to exclude all charge states for a specified precursor. For turboDDA, no dynamic exclusion was used and top 100 MS/MS candidate ion scans were acquired. The collection of MS2 spectra was completed in the linear ion

trap (isolation window: 1.6 m/z; scan rate: rapid; AGQ target: standard; MaxIT: Auto; HCD CE: 35%; data type: centroid).

Cell lysate digests from Calu-6 and A549 were analyzed with a nanoAcquity UPLC system (Waters) coupled with TripleTOF 6600 mass spectrometer (AB Sciex). Samples were resuspended in 40 µL 0.1% FA solution and loaded onto a C18 Trap column (Waters Acquity UPLC M-Class Trap, Symmetry C18, 100 Å, 5 µm, 180 µm × 20 mm) at 10 µL/min for 4 min. Peptides were then separated with an analytical column (Waters Acquity UPLC M-Class, peptide BEH C18 column, 300 Å, 1.7 µm, 75 µm × 150 mm) which was temperature controlled at 40 °C. The flow rate was set as 400 nL/min. A 120-min gradient of buffer A (2% ACN, 0.1% formic acid) and buffer B (0.1% formic acid in ACN) was used for separation: 1% buffer B at 0 min, 5% buffer B at 1 min, 30% buffer B at 60 min, 50% buffer B at 90min, 50% buffer B at 100 min, 98% buffer B at 105 min, 98% buffer B at 100 min, 1% buffer B at 100.1 min, and 1% buffer B at 120 min. Data were acquired with the mass spectrometer using an ion spray voltage of 2.3 kV, GS1 5 psi, GS2 0, CUR 30 psi and an interface heater temperature of 150 °C. Mass spectra was recorded with Analyst TF 1.7 software in the DDA mode. The data without dynamic exclusion (turboDDA) was obtained with the set of never exclude former target ions and precursors that exceeded a threshold of 100 cps were selected for $MS^2$ and 100 × 30 ms $MS^2$ candidate ion scans from 100–1800 Da were acquired in high sensitivity mode. Adjusted CE when iTRAQ reagent was used.

**Data analysis for 1 ng, 10 ng and 100 ng of K562 digests acquired on SCIEX Triple TOF instrument**

Mass spectrometry data generated were stored, searched, and analyzed using the ProHits laboratory information management system platform.[3] Within ProHits, WIFF files were converted to an MGF format using the WIFF2MGF converter and to a mzML format using ProteoWizard (V3.0.10702) and the AB SCIEX MS Data Converter (V1.3 beta). The data were then searched using Mascot (V2.3.02) and Comet (V2016.01 rev.2). The spectra were searched against the human and adenovirus sequences in the RefSeq database (version 57, January 30th, 2013) acquired from NCBI, supplemented with "common contaminants" from the Max Planck Institute (http://lotus1.gwdg.de/mpg/mmbc/maxquant_input.nsf/7994124a4298328fc125748d0048fee2/$FILE/contaminants.fasta) and the Global Proteome Machine (GPM; https://www.thegpm.org/crap/), forward and reverse sequences (labeled "gi|9999" or "DECOY"), sequence tags (BirA, GST26,

mCherry, and green fluorescent protein (GFP)) and streptavidin, for a total of 72,481 entries. Database parameters were set to search for tryptic cleavages, allowing up to two missed cleavage sites per peptide with a mass tolerance of 35 ppm for precursors with charges of 2+ to 4+ and a tolerance of 0.15 amu for fragment ions. Deamidated asparagine and glutamine and oxidized methionine were selected as variable modifications. Results from each search engine were analyzed through the Trans-Proteomic Pipeline (v.4.7 POLAR VORTEX rev 1) via the iProphet pipeline.[4]

**Data analysis for 1 ng and 10 ng of K562 digests acquired on a Thermo Orbitrap Eclipse instrument**

Proteome Discoverer Software (version 2.5, San Jose, CA) was used to process raw files for detecting features, searching databases and quantifying proteins/peptides. The search of MS/MS spectra was conducted against the UniProt human database (downloaded on June 27$^{th}$, 2022, containing 79,435 entries). Methionine oxidation and N-terminal protein acetylation were chosen as variable modifications, while the carbamidomethylation of cysteine residues was regarded as a fixed modification. Precursors and fragments had a mass tolerance of 10 ppm and 0.6 Da, respectively. Minimum and maximum peptide lengths were six and 144 amino acids, respectively. The missed cleavage allowed for every peptide was two. The filtering of proteins had a maximum false discovery rate (FDR) of 0.01. The default settings of Proteome Discoverer were other parameters that were not mentioned.

**MaxQuant database searching for iTRAQ labeled three proteome mixture samples**

For protein identification and quantification of iTRAQ labeled three proteome mixture sample, the wiff files from the Sciex TripleTOF 6600 system were imported into MaxQuant (version 1.6.3.4) with integrated Andromeda database search engine. The MS/MS spectra were queried against the fasta file combined by three databases from human (65,536 entries), yeast (6,068 entries) and *E. coli* (4,306 entries). Database search employed the following parameters: Reporter ion MS2 with multiplicity 8plex for the iTRAQ 8-plex experiments, trypsin digestion with maximum 2 missed cleavages, oxidation of methionine and acetylation of protein N-termini as variable modifications, carbamidomethylation of cysteine as fixed modification, maximum number of modifications per peptide set at 5, minimum peptide length of 6, and protein FDR 0.01. The precursor intensity filtering (PIF) was set to 0, 0.25, 0.5 or 0.75 when testing their effects on identification and quantification. Instrument type was set to AB SCIEX Q-TOF and the isolation window used in

PIF analysis would be automatically changed according to the isolation window setting in the raw files that loaded onto MaxQuant.[1] Appropriate correction factors for the individual iTRAQ channels for both peptide N-terminal labelling and lysine side-chain labelling as per the iTRAQ Reagent Multiplex Kit were also configured into the database search.

Data files from A549 and Calu-6 cells were submitted for simultaneous searches using ProteinPilot version 5.0 software (Sciex) utilizing the Paragon and Progroup algortihms and the integrated false discovery rate (FDR) analysis function. MS/MS data was searched against either the Uniprot human database (reviewed, 20,353 entries) downloaded on 20200720. Trypsin was selected as the enzyme. Fixed modifications of carbamidomethylation at Cys and iTRAQ 8-plex at Lys and the N-terminal amino group of peptides were set. Other search parameters include instrument (TripleTOF 6600), ID Focus (Biological modifications), search effort (Thorough), false discovery rate (FDR) analysis (Yes), and user modified parameter files (No). The proteins were inferred based on the ProGroupTM algorithm associated with the ProteinPilot software. The detected protein threshold in the software was set to the value which corresponded to 1% FDR. Peptides were defined as redundant if they had identical cleavage site(s), amino acid sequence, and modification.

## References


(1) Tyanova, S.; Temu, T.; Cox, J. The MaxQuant Computational Platform for Mass Spectrometry-Based Shotgun Proteomics. *Nat Protoc* **2016**, *11* (12), 2301–2319. https://doi.org/10.1038/nprot.2016.136.

(2) Wu, C.; Zhou, S.; Mitchell, M. I.; Hou, C.; Byers, S.; Loudig, O.; Ma, J. Coupling Suspension Trapping–Based Sample Preparation and Data-Independent Acquisition Mass Spectrometry for Sensitive Exosomal Proteomic Analysis. *Anal Bioanal Chem* **2022**, *414* (8), 2585–2595. https://doi.org/10.1007/s00216-022-03920-z.

(3) Liu, G.; Zhang, J.; Larsen, B.; Stark, C.; Breitkreutz, A.; Lin, Z.-Y.; Breitkreutz, B.-J.; Ding, Y.; Colwill, K.; Pasculescu, A.; Pawson, T.; Wrana, J. L.; Nesvizhskii, A. I.; Raught, B.; Tyers, M.; Gingras, A.-C. ProHits: Integrated Software for Mass Spectrometry-Based Interaction Proteomics. *Nat Biotechnol* **2010**, *28* (10), 1015–1017. https://doi.org/10.1038/nbt1010-1015.

(4) Shteynberg, D.; Deutsch, E. W.; Lam, H.; Eng, J. K.; Sun, Z.; Tasman, N.; Mendoza, L.; Moritz, R. L.; Aebersold, R.; Nesvizhskii, A. I. iProphet: Multi-Level Integrative Analysis of Shotgun Proteomic Data Improves Peptide and Protein Identification Rates and Error Estimates. *Mol Cell Proteomics* **2011**, *10* (12), M111.007690. https://doi.org/10.1074/mcp.M111.007690.

**Table S1.** Optimization of acquisition parameters for the turboDDA and DEDDA methods (10 ng or 1 ng of the K562 digest was used as standard for testing; n = 2).

| Method | Sample amount | Dynamic exclusion | Q1 resolution | Top MS/MS candidate number | Precursor intensity threshold | MS/MS accumulation time (ms) | Proteins(#) | Peptides(#) |
|---|---|---|---|---|---|---|---|---|
| DEDDA | 10ng | 7s after 1 time | Unit | 10 | 300 cps | 100 | 585 | 2095 |
| DEDDA 20s dynamic exclusion time | 10ng | 20s after 1 time | Unit | 10 | 300 cps | 100 | 482 | 1592 |
| DEDDA after 3 times | 10ng | 7s after 3 times | Unit | 10 | 300 cps | 100 | 544 | 1991 |
| DEDDA Top 100 MS/MS | 10ng | 7s after 1 time | Unit | 100 | 300 cps | 30 | 537 | 1925 |
| turboDDA | 10ng | no | Unit | 100 | 100 cps | 30 | 1073 | 3905 |
| turboDDA 30cps | 10ng | no | Unit | 100 | 30 cps | 30 | 677 | 2182 |
| turboDDA 45ms | 10ng | no | Unit | 100 | 100 cps | 45 | 933 | 3681 |
| turboDDA low resolution Q1 | 10ng | no | Low | 100 | 100 cps | 30 | 934 | 3337 |
| turboDDA Top 50 MS/MS 60ms accumulation time | 10ng | no | Unit | 50 | 100 cps | 60 | 959 | 3430 |
| DEDDA | 1ng | 7s after 1 time | Unit | 10 | 300 cps | 100 | 25 | 58 |
| turboDDA | 1ng | no | Unit | 100 | 100 cps | 30 | 146 | 299 |

**Table S2.** Acquired total MS/MS spectra for turboDDA and DEDDA (n = 3).

| | turboDDA | DEDDA |
|---|---|---|
| 100 ng K562 digests | 173836±1325 | 34900±4793 |
| 10 ng K562 digests | 92235±3006 | 13507±156 |

**Table S3.** The number of proteins identified from 250 ng and 25 ng of K562 cell digests analyzed by turboDDA and DEDDA methods.

| | 250 ng sample | | 25 ng sample | |
|---|---|---|---|---|
| | turboDE high pH frac | DE-DDA high pH frac | turboDE high pH frac | DE-DDA high pH frac |
| Protein | 5459 | 4609 | 3794 | 2792 |
| Peptide | 43742 | 32687 | 15780 | 13165 |
| PSM | 350222 | 148347 | 95831 | 48364 |

**Figure S1.** A three-proteome model to simulate complex samples. (A) Schematic of reporter ion intensities for samples consisting of six iTRAQ channel labeled yeast digests, four iTRAQ channel labeled human cell digests, and four iTRAQ channel labeled *E. coli* digests. (B) An ideal yeast peptide $MS^2$ spectrum without human and *E. coli* peptide interference in the first three channels would have identical and mirrored iTRAQ reporter ion intensities in the last three channels. (C) Typical yeast peptide spectra have some interference from human (red) or/and *E. coli* (blue) peptide, resulting in a ratio distortion toward 10:6:1.

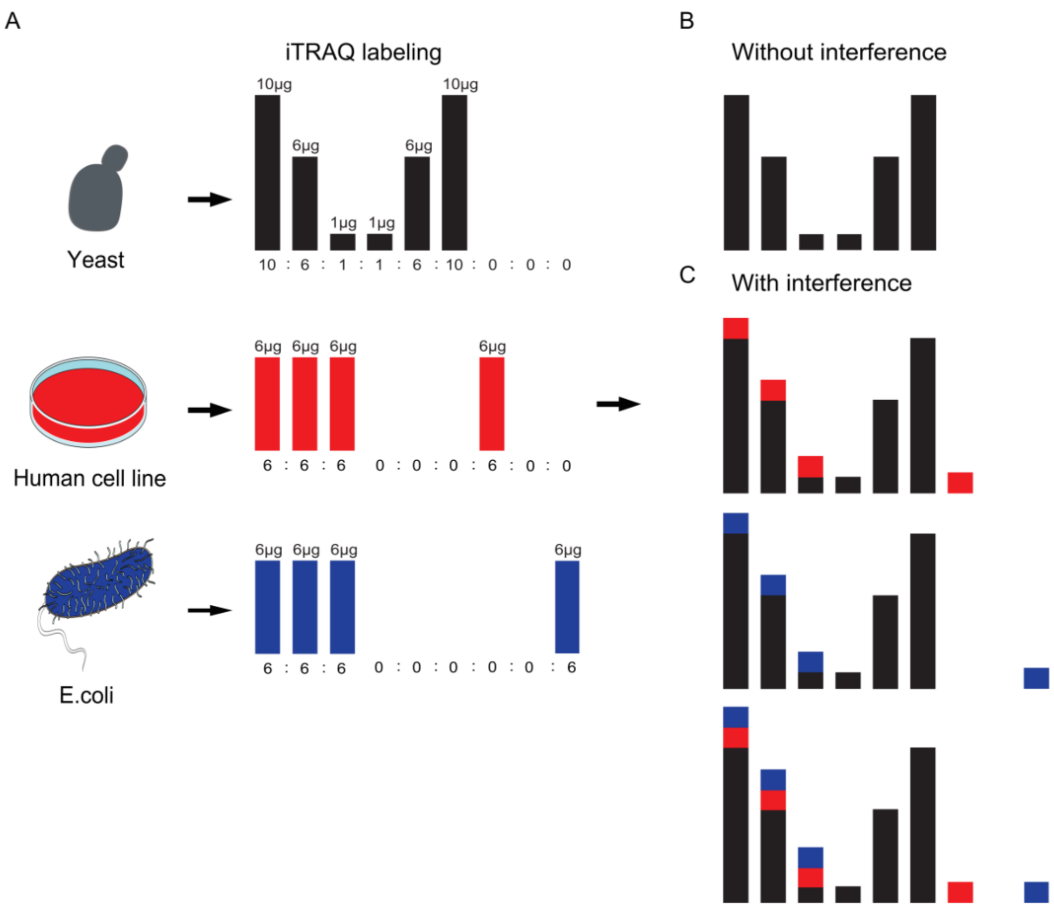

**Figure S2.** Venn diagrams on protein (A and C) and peptide (B and D) level for the combined results of three replicate of turboDDA and DEDDA.

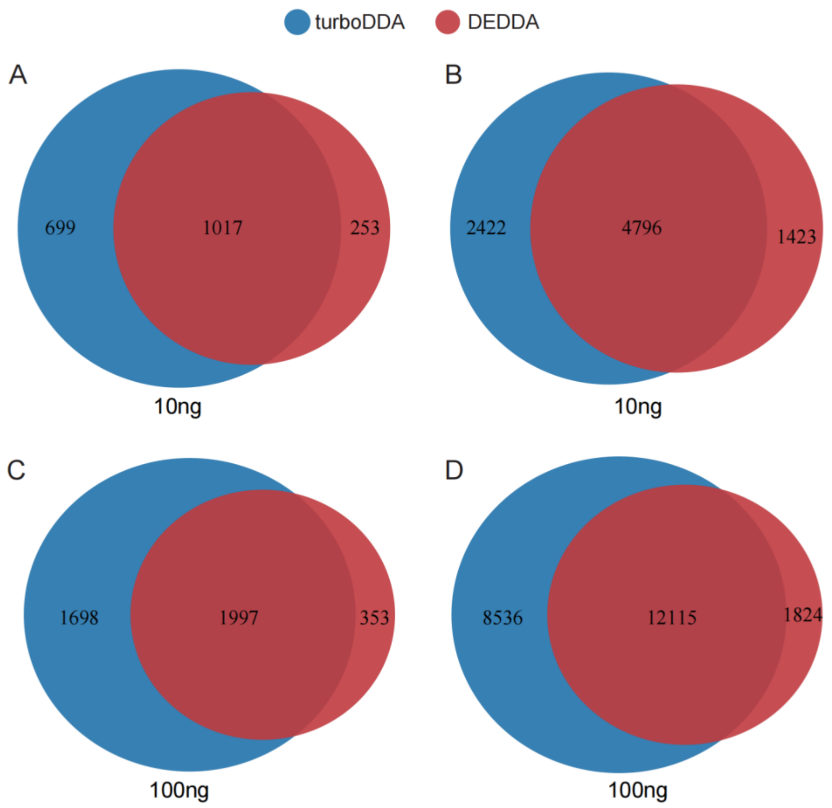

**Figure S3.** Acquired MS/MS spectra and identified MS/MS spectra for DEDDA (A and B) and turboDDA (C and D) along retention time.

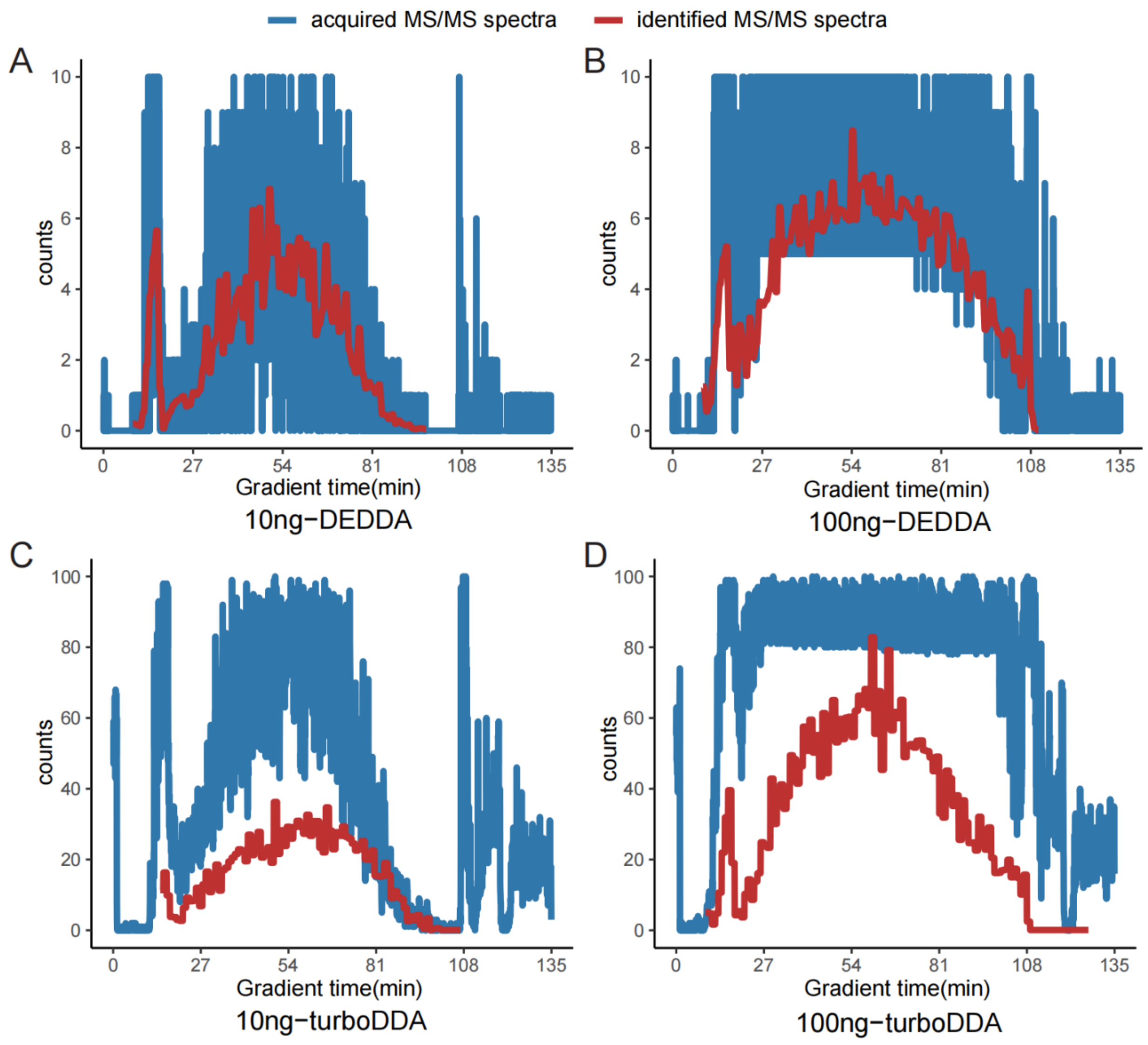

**Figure S4.** Grand average of hydropathy (GRAVY) value (A and B), isoelectric point (pI) (C and D) and molecular weight (MW) (E and F) distribution of the peptides identified by turboDDA and DEDDA for 10 ng and 100 ng K562 digest; and the abundance distribution for the commonly identified and unique identified proteins by turboDDA and DEDDA for 10ng (G) and 100ng (H) K562 digest.

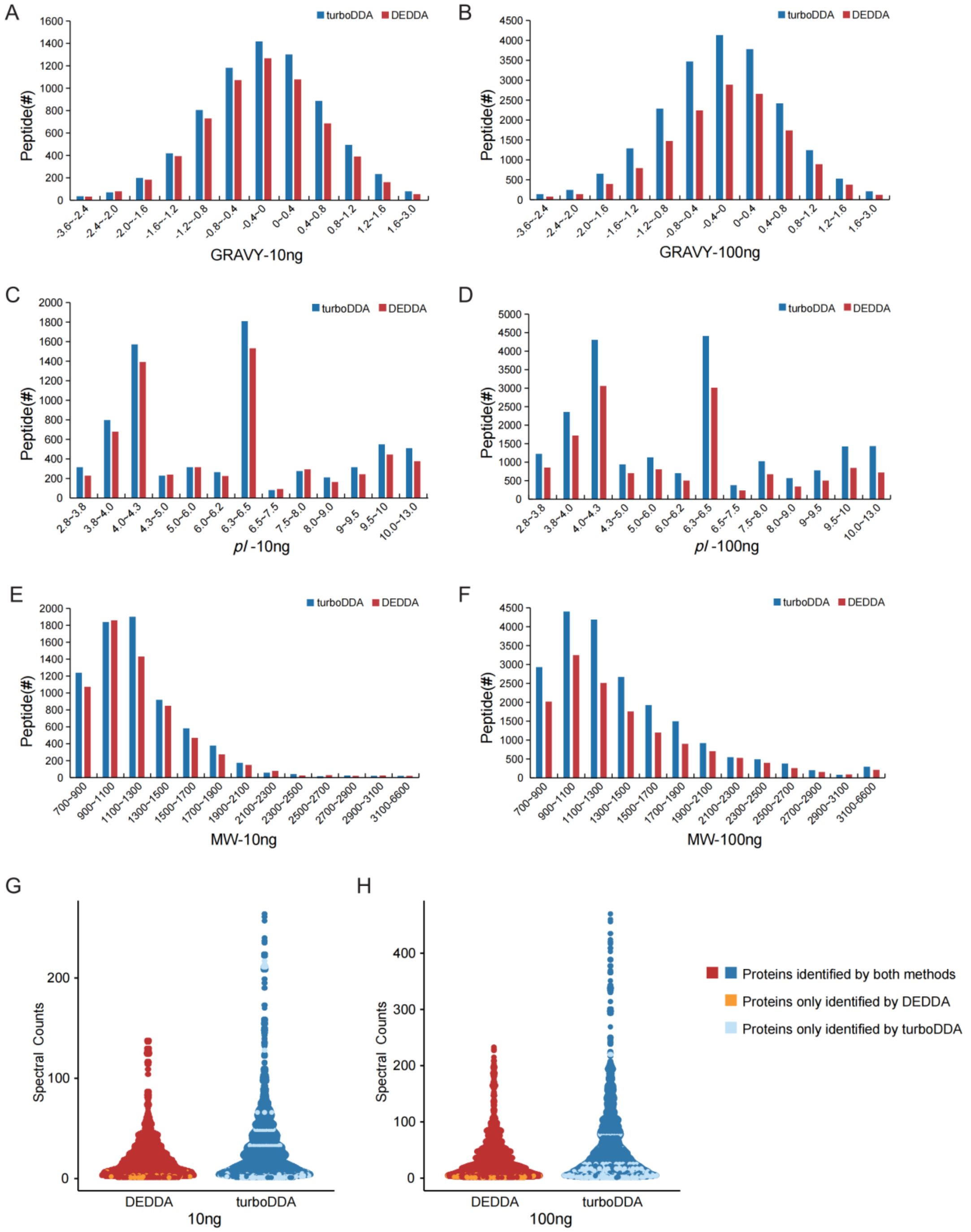

**Figure S5.** Typical spectra of peptides QQPGPSEHIER, VTVMLYDDGNK and VNDFLAEIFK from proteins VASP (P50552, Vasodilator-timulated phosphoprotein) and CKAP5 (Q14008, Cytoskeleton-associated protein 5) acquired by turboDDA (A, C and E) and DEDDA (B, D and F).

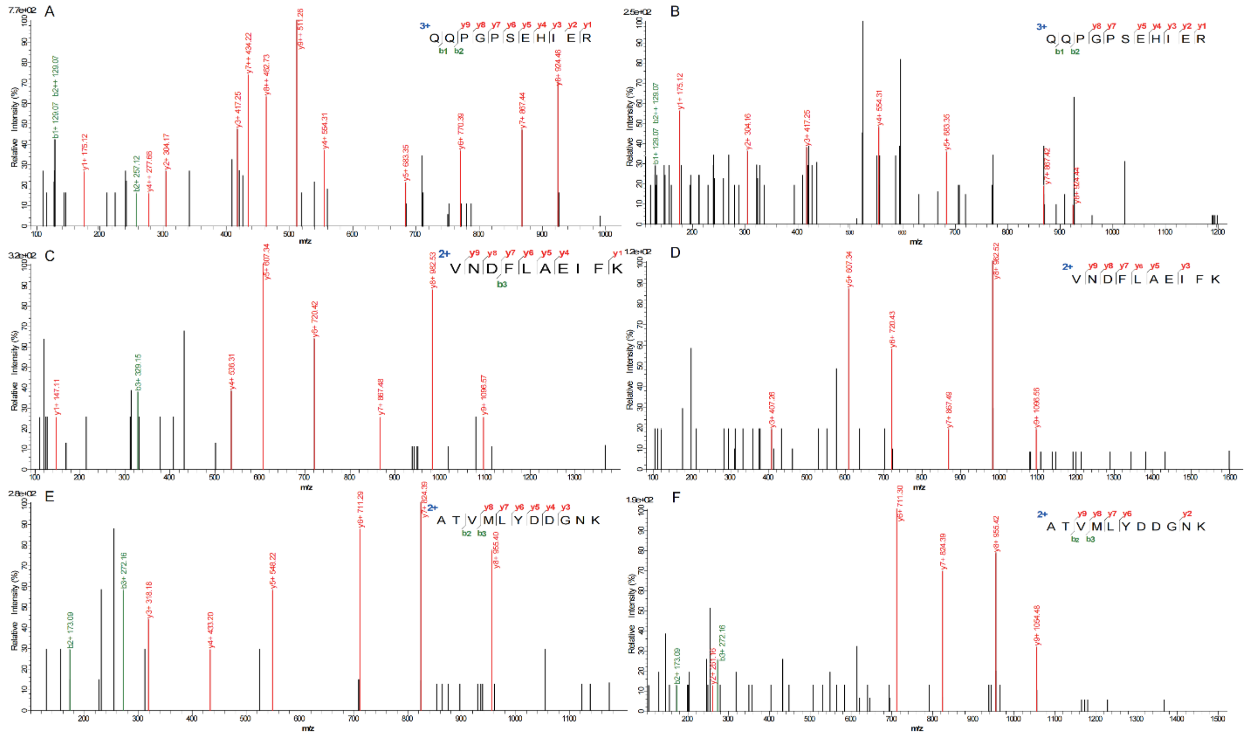

**Figure S6.** Trace amount of sample analysis by turboDDA and DEDDA on Thermo Orbitrap Eclipse instrument. The number of proteins and peptides identified from 1 ng (A and B) and 10 ng (C and D) of human K562 digests analyzed by turboDDA and DEDDA methods (*n*=3, average). $^*p < 0.05$, $^{**}p < 0.01$, and $^{***}p < 0.001$, by two-tailed unpaired Student's *t*-test. The coefficients of variation (CV) distribution of LFQ intensities from three injections of turboDDA and DEDDA for 1 ng (E) and 10 ng (F) K562 digests.

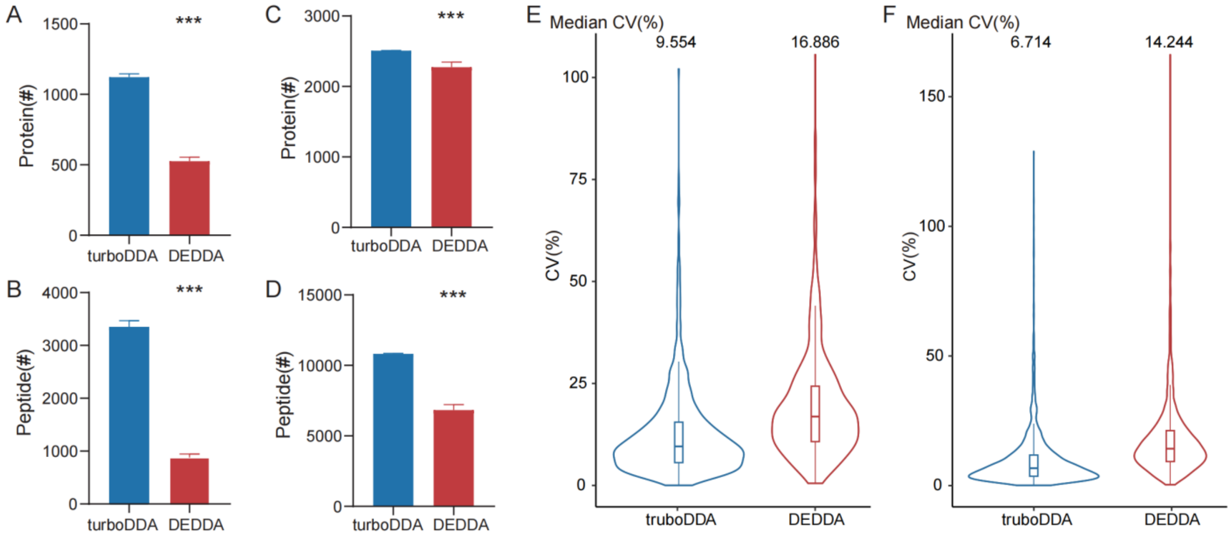

**Figure S7.** PIF value distribution of iTRAQ labeled three proteome mixtures for turboDDA and DEDDA.

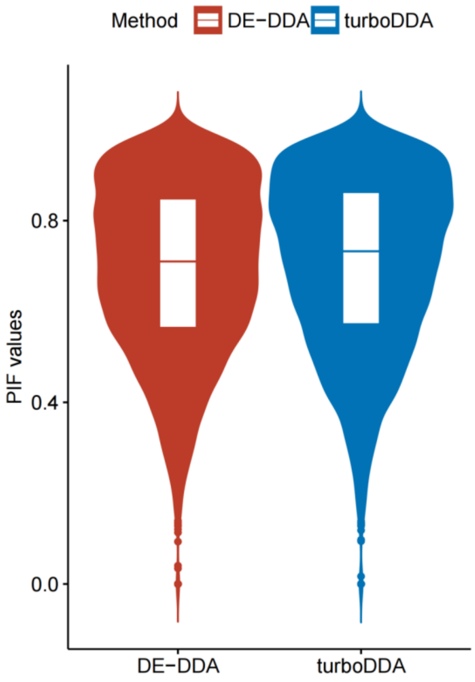

**Figure S8.** Impact of PIF on interference free index (IFI) distribution and quantified peptide number for turboDDA and DEDDA. (A) Equation for calculating the interference-free index (IFI). (B) IFI distribution under different PIF cutoff. (C) Quantified yeast peptide number under different PIF cutoff.

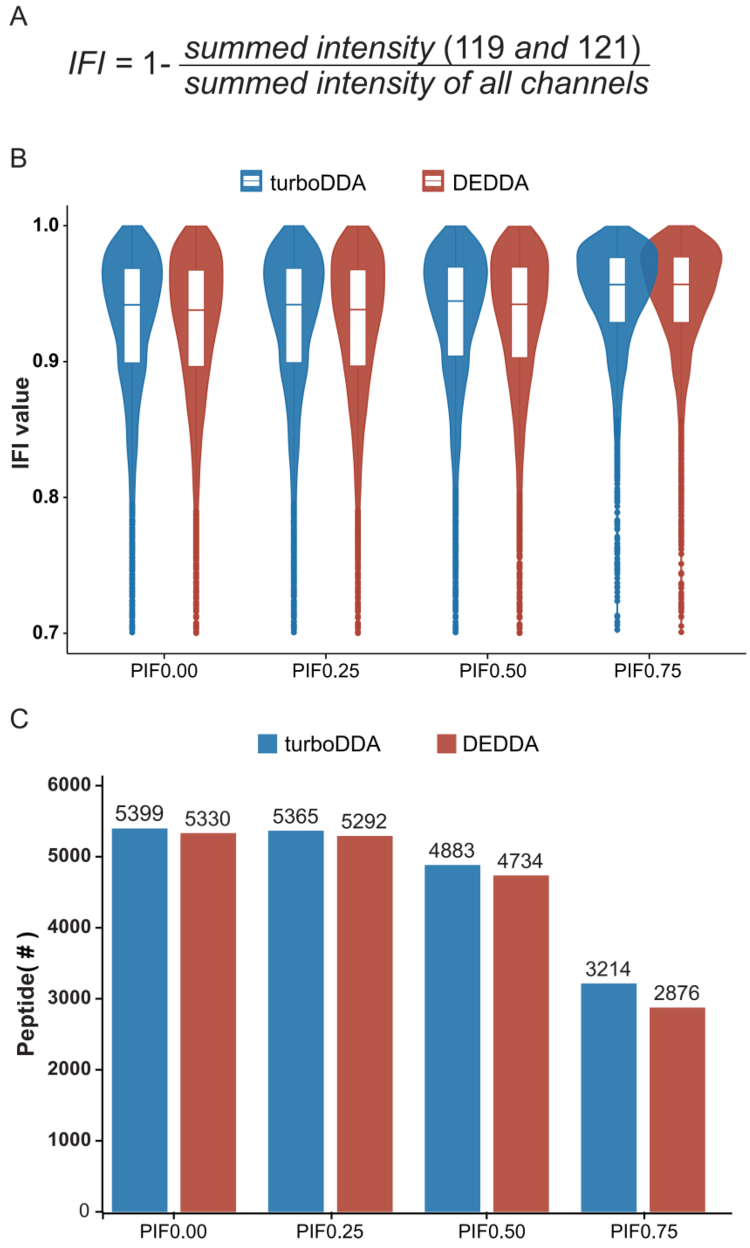

**Figure S9.** GO analysis of the molecular functions (A) and the interaction network (B) of differential proteins from lung cancer cell lines.

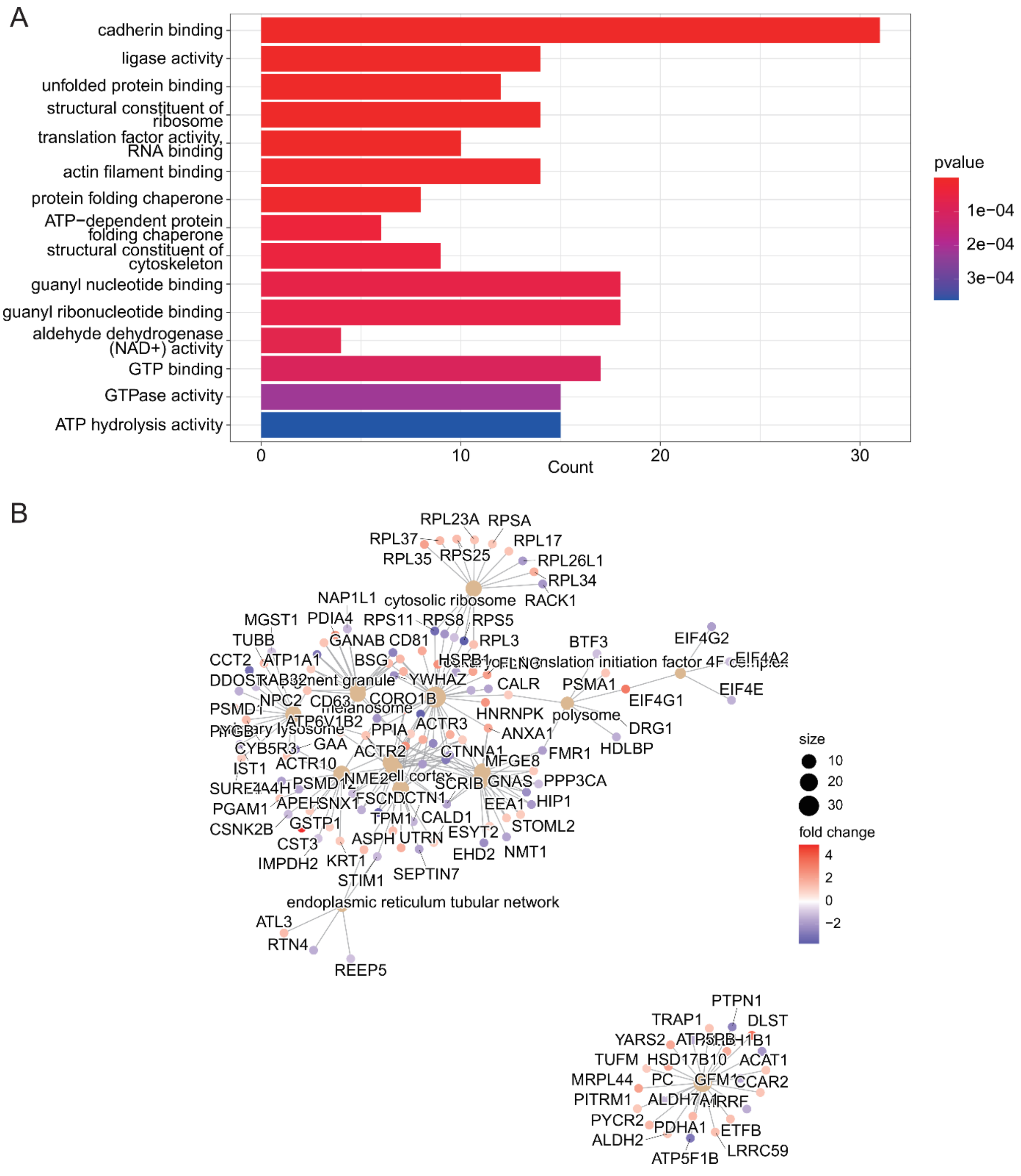

Supplement: Supplementary file 1 — ac3c03357_si_001.pdf [file ac3c03357_si_001.pdf]
